# Climate change-induced water stress suppresses the regeneration of the critically endangered forest tree *Nyssa yunnanensis*

**DOI:** 10.1371/journal.pone.0182012

**Published:** 2017-08-01

**Authors:** Shanshan Zhang, Hongmei Kang, Wenzhong Yang

**Affiliations:** Yunnan Key Laboratory of Forest Plant Cultivation and Utilization, State Forestry Administration Key Laboratory of Yunnan Rare and Endangered Species Conservation and Propagation, Yunnan Academy of Forestry, Kunming, Yunnan, China; Odum School of Ecology, University of Georgia, UNITED STATES

## Abstract

Climatic change-induced water stress has been found to threaten the viability of trees, especially endangered species, through inhibiting their recruitment. *Nyssa yunnanensis*, a plant species with extremely small populations (PSESP), consists of only two small populations of eight mature individuals remaining in southwestern China. In order to determine the barriers to regeneration, both *in situ* and laboratory experiments were performed to examine the critical factors hindering seed germination and seedling establishment. The results of *in situ* field experiments demonstrated that soil water potentials lower than -5.40 MPa (experienced in December) had significantly inhibitory effects on seedling survival, and all seedlings perished at a soil water potential of -5.60 MPa (January). Laboratory experiments verified that *N*. *yunnanensis* seedlings could not survive at a 20% PEG 6000 concentration (-5.34 MPa) or 1/5 water-holding capacity (WHC; -5.64 MPa), and seed germination was inhibited in the field from September (-1.10 MPa) to November (-4.30 MPa). Our results suggested that soil water potentials between -5.34 and -5.64 MPa constituted the range of soil water potentials in which *N*. *yunnanensis* seedlings could not survive. In addition to water deficit, intensified autotoxicity, which is concentration-dependent, resulted in lower seed germination and seedling survival. Thus, seed establishment was probably simultaneously impacted by water deficit and aggravated autotoxicity. Meteorological records from the natural distribution areas of *N*. *yunnanensis* indicated that mean annual rainfall and relative humidity have declined by 21.7% and 6.3% respectively over past 55 years, while the temperature has increased by 6.0%. Climate change-induced drought, along with a poor resistance and adaptability to drought stress, has severely impacted the natural regeneration of *N*. *yunnanensis*. In conclusion, climate change-induced drought has been implicated as a regulating factor in the natural regeneration of *N*. *yunnanensis* through suppressing seed germination and screening out seedlings in the dry season. Based on the experimental findings, habitat restoration and microclimate improvement should both be highlighted in the conservation of this particular plant species.

## Introduction

Global climatic change-induced drought stress has manifested significant influences on forest regeneration in many regions of the world [1–4]. In recent decades, changes in the global climate as a result of increasing greenhouse gas emissions have resulted in drought and/or higher temperatures [5]. Furthermore, the frequency and intensity of droughts is increasing [6]. In fact, increased drought has already resulted in ecological change in many forest communities [7–8], and drought-related forest regeneration failure has been well documented [1,9–13]. As suggested by Bartholomeus et al. [14], water-related stresses have more severe effects on the future distributions of endangered plant species, as these species usually have restricted distributions and do not possess the physiological and morphological adaptations to prevent excessive water loss. Furthermore, plants in early life stages are more sensitive to changes in climate, which can present a major bottleneck to recruitment [2,15–16].

Seed germination and seedling establishment are two critical early life stages in which drought stress affects forest regeneration [17–20]. Increases in temperature as well as moisture limitation may make species particularly susceptible to climate change during these life stages. For many plant species, constraints experienced during the phases of seed germination and seedling establishment severely limit regeneration [15–16,21]. The fate of seedlings has been found to be closely associated with precipitation, which is often highly seasonal [22]. Studies on woody plants have indicated that the length of the dry season affects seedling growth and survival, and continuous precipitation is also favorable for seedling growth [22–24]. Additionally, seed germination and early seedling establishment depend primarily on water availability, and increased germination rates are correlated with higher rainfall in some species [25–26]. Thus, studies on the effects of drought on plant regeneration may provide insights into population dynamics, mechanisms of survival as well as inform conservation strategies [27–28].

*Nyssa yunnanensis* is a critically endangered and range-restricted tree species known only from two small populations, consisting of five and three mature individuals respectively. It distributed in ravine rainforest and remained in the tropical forest region of southern Yunnan Province, southwestern China [29–30]. It has been delineated on the China's Flora Red List [31] and the IUCN Red List as a Critically Endangered Species. Although *N*. *yunnanensis* naturally produces abundant seeds, no seedlings were found surrounding the mother trees upon inspection of the locality. Chen et al. [32] suggested that difficulties in seed germination and seedling growth in wild populations of *N*. *yunnanensis* hamper its regeneration. Studies on the taxonomy, morphology, seed germination characteristics and reproductive biology of *N*. *yunnanensis* [33–36] have not identified any physiological constraints to regeneration. However, Zhang et al. [30] proposed that regeneration in *N*. *yunnanensis* may be negatively impacted by its autotoxicity. Moreover, autotoxic effects were found to be concentration-dependent, such that rainfall and soil moisture availability becomes a significant influencing factor. Significantly, meteorological records since 1960 have indicated that annual precipitation in the natural distribution areas of *N*. *yunnanensis* has decreased significantly during the past half century [30]. However, the impacts of drought stress on the suppression of *N*. *yunnanensis* regeneration and survival under climate change are not currently known.

Using field surveys and observations, we designed crosschecking experiments in order to examine the impact of drought as a regulating factor in the natural regeneration of *N*. *yunnanensis*. *In situ* experiments were designed to examine whether water acted as a vital factor in both seed germination and seedling survival. Laboratory experiments were then performed to verify the observations from the *in situ* field experiments. In the laboratory experiments, the seedlings were measured at two development stages, i.e., early seedling and post-seedling, in order to examine how drought stress influences the entire seedling establishment process.

## Material and methods

### Study site and climatic data

*Nyssa yunnanensis* occurs in the natural forests of Puwen Forest Farm (22°25′N, 101°05′E), Yunnan Province, southwestern China. In accordance with the seasonal changes in temperature and rainfall, three seasons in a year were defined for the study site, i.e., rainy season, fog cool season and hot season. The rainy season is from May to October and experiences a mean annual rainfall of 1167 mm; the fog cool season lasts from November to February and experiences less rainfall (110 mm per annum); the hot season is from March to April and is associated with elevated temperatures and the least rainfall (73 mm per annum) [37]. Seed germination and seedling establishment of *N*. *yunnanensis* occurs from September to February, during the latter part of the rainy season and the entire fog cool season.

We obtained monthly climatic data including mean temperature, precipitation and relative humidity from meteorological stations located near the study sites for the period of 1960–2014 (S1 Fig and S2 Fig).

### Materials

Mature seeds of *Nyssa yunnanensis* were harvested from both remaining populations in August 2013 from their native habitat in Puwen Forest Farm,Yunnan Academy of Forestry. They were stored in a cold room (4°C) after drying at room temperature (20°C). In order to exclude the autotoxic effects of its pericarp, a seed-washing treatment was employed to break dormancy [30]. Before sowing, the seeds were selected based on size homogeneity and surface-sterilized to prevent decay by soaking in 5% Na-hypochlorite for five min, followed by washing five times with distilled water. We used 50 post seedlings in the follow-up experiments. They were cultivated for five weeks in a growth chamber (Safe Experimental Instrument Company, Haishu, Ningbo, China) at 20/15°C (day/night), under a 14-h photoperiod and a light intensity of 100–110 μmol m^−2^ s^−1^ and 90% relative humidity. The seedlings were then transplanted into pots containing sterilized bed soil (60% vermiculite, 20% cocopeat and 20% of other additives such as zeolite, loess and peat moss), and were placed into regular microcosm pots (10 cm dia. × 7 cm depth) in a greenhouse.

### Methods

#### *In situ* field experiment

The aim of the field experiment was to examine seed germination and seedling emergence of *N*. *yunnanensis* in *in situ* soil cultures within their natural environment. A 1-factorial experiment was designed consisting of two treatments: sowing in pots, and sowing directly in the soil in the field. In the “pot” treatment, five microcosm pots (30 cm dia. ×25 cm dep.) containing 5 kg of *in situ* soil were placed under the canopy of each target mother tree and treated with sufficient moisture artificially in the morning every two days. A 0.8 m tall fence was installed around the perimeter of the plot to deter entry and subsequent trampling. In the”field” treatment, five furrows were dug (45 cm long × 20 cm wide) in the field close to the pots, and received only natural moisture. Three *N*. *yunnanensis* mother trees bearing fruits were used for the study. In total, there were 15 replicates for the three target mother trees in each treatment. No additional nutrients were added to either treatment. One hundred seeds were sown in each plot. Seed germination rate, seedling emergence, seedling survival and soil water potential (using a soil moisture sensor: Watermark, Spectrum, USA) were recorded monthly during the experimental period. The equation to calculate germination rate is: GR = seeds germinated /total seeds × 100.

#### Laboratory experiments

*Experiment 1*: *Effects of simulated drought stress (SDS) on seed germination and early seedling growth*: This experiment was carried out to determine the effects of simulated drought stress on germination and early seedling growth. Osmotic solutions of polyethylene glycol (PEG 6000) were used to induce drought stress reproducibly under *in vitro* conditions, and to maintain consistent water potential throughout the experimental period. The solutions were prepared by dissolving the required amount of PEG 6000 in distilled water and shaking in a shaker bed (at 25°C) for 12 h.

Seed germination and early seedling growth were evaluated under five concentrations of PEG 6000 treatment (5%, 10%, 15%, 18% and 20%), corresponding to water potentials of -0.58, -1.66, -3.25, -4.29 and -5.34 MPa, respectively, while distilled water was used as the control (-0.01 MPa). The experiment was performed in Petri dishes (9 cm diameter) in a greenhouse. A 1-factorial seed germination experiment was designed with five PEG 6000 concentrations including six replicates, resulting in a total of 30 Petri dishes.

The seeds were sterilized by soaking in a 5% solution of H_2_O_2_ for five min. After the treatment, the seeds were washed several times with distilled water. Thirty seeds based on size homogeneity were placed on filter paper moistened with the respective PEG 6000 solutions in each 9 cm diameter Petri dish. The Petri dishes were covered with parafilm to prevent the loss of moisture by evaporation. The dishes were then placed in an incubator for 40 days at 25°C. After 40 days of incubation, seed germination and early seedling growth indicators including shoot length, root length, seedling length and seedling fresh weight were measured. Seeds were considered germinated when the emergent radical reached 2 mm in length. Time to 50% germination (time taken to reach 50% germination) was calculated referred to Cavallaro et al. [38]. Germination rate (GR) was calculated using the formula: GR = seeds germinated/total seeds × 100.

*Experiment 2*: *Effects of watering treatments on post-seedling growth*: A water-controlled experiment was conducted to study the effects of simulated water conditions corresponding to various soil moistures on seedling growth. In this experiment, one-year-old seedlings were subsequently subjected to five soil moisture levels: 1 WHC (water holding capacity), 1/2 WHC, 1/3 WHC, 1/4 WHC and 1/5 WHC encompassing the range of soil moistures experienced in the field. The corresponding soil water potentials were determined using the filter paper technique [39], and were determined to be -0.01, -1.18, -2.30, -4.46 and -5.64 MPa, respectively. Indicators of seedling growth were measured at the end of experiment.

Intact forest soils from the natural habitat of *N*. *yunnanensis* were used in this experiment to assess seedling performance across a gradient of soil moisture contents. At the field site, 30 soil cores (10 cm in diameter and 15 cm in height) in three sampling areas (2 m × 2 m) were collected from the rhizospheres of *N*. *yunnanensis*. Soil cores were transported to the laboratory immediately. Each soil core was transferred into a microcosm pot (10 cm diameter × 7 cm depth).

The pots were arranged in a greenhouse and positioned in a way that they would have likely received uniform amounts of sunlight, amount of shading, etc. Seedlings were irrigated daily with deionized water. Annual seedlings used in the experiment were propagated. All of the seedlings received watering for approximately one month prior to the initiation of experiments in order to help them acclimatize, as well as to minimize initial mortality. Desired soil moisture levels were obtained by allowing the soil to dry until it approached the selected moisture level, as determined gravimetrically on a parallel set of identical pots maintained for this purpose in the glasshouse (Khurana and Singh, 2004). The pots were weighed on alternate days and the required amount of water was added in order to maintain the respective moisture levels as accurately as possible. The plants were harvested following 90 days of growth. The aboveground organ of each plant was cut at the soil level and dried at 65°C until a constant mass was achieved, and then biomass was determined by weighing. Pots were placed at 4°C until roots could be washed (within one week). Belowground mass was separated from the growing media by washing with water, and dried at 65°C until a constant mass was reached. Growth measurements were determined as follows: leaf area ratio (LAR) = total leaf area/ total biomass; specific leaf area (SLA) = total leaf area/ total leaf biomass.

### Statistical analyses

A repeated-measure one-way ANOVA (SPSS V. 16.0) was performed to determine the effect of sowing treatments in *in situ* experiments. Data obtained from the laboratory experiments, including seed water absorption, germination rate, early seedling growth and post seedling growth were evaluated using one-way ANOVA. If ANOVAs were significant, LSD was used to separate means at the 5% level. As the early seedlings in Experiment 1 did not survive under the treatment with 20% PEG 6000 solution (-5.34 MPa), and similarly, the post-seedlings in Experiment 2 did not survive under the 1/5 WHC (-5.64 MPa) treatment, they were excluded from the above analyses.

## Results

### Seed germination

**Fig 1:**
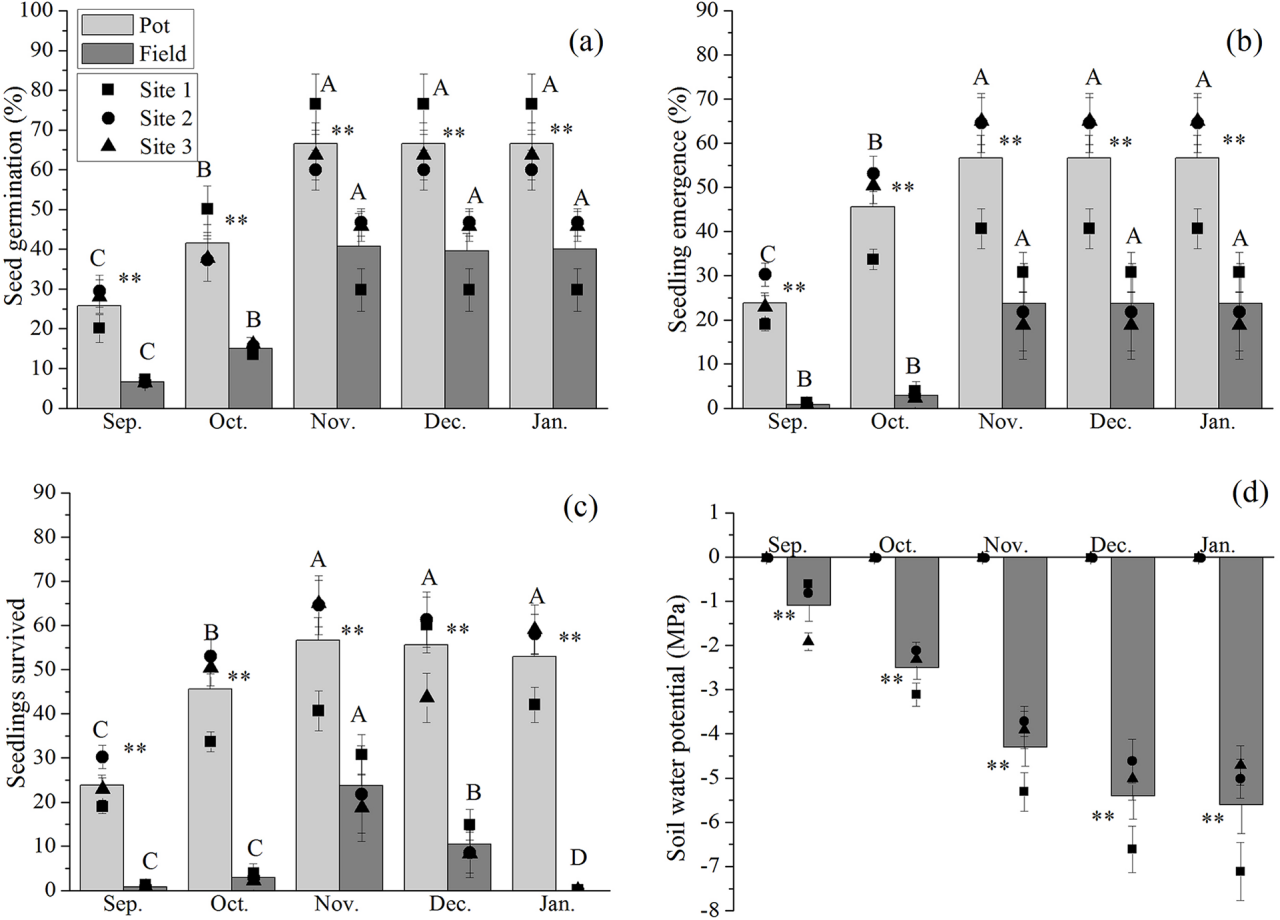
Seed germination (a), seedling emergence (b), seedling survived (c) and soil water potential (d) for *Nyssa yunnanensis* in pots with sufficient moisture, and in field with natural moisture at three sites during the entire seedling establishment period. Squares, circles and triangles represent site means; values are means ± SD; ** indicates a significant difference between treatments in “Pot” and “Field” at *P* < 0.01; For the soil with sufficient moisture (Pot) or natural moisture (Field), means with different uppercase are significantly different from September to January at *P* < 0.05.

Results of the *in situ* field experiments revealed that the rate of seed germination in *N*. *yunnanensis* was significantly different between the sowing treatments (pot and field) and months (from September to January; Table 1). Seed germination began one week after sowing in September and was determined to be 25.81% in the pots and 6.64% in the field (Fig 1A). It increased with time and reached its highest value in November. The highest germination rate in the pots was 66.61%, while in the field it was 40.69%. During the following two months there were no significant changes in both treatments. This suggests that seed germination terminates at the end of November under conditions of both sufficient and natural moisture. Seed germination in the pots was significantly higher than that in the field during the germination period (*P* < 0.01); soil water potential in the pots (-0.01 MPa) was kept in saturation state during the entire period of seed germination, while in the field it decreased progressively i.e., -1.10 MPa in Sep., -2.50 MPa in Oct., -4.30 MPa in Nov., -5.40 MPa in Dec. and -5.60 MPa in Jan.

**Table 1:** Significance level of the effects of factors and factor interactions on variables based on repeated-measure one-way ANOVA.

Variables	df	Seed germination	Seedling emergence	Seedlings survived	Soil water potential
Sowing treatment	1	**(*F* = 895.957)	**(*F* = 628.606)	**(*F* = 1.636E3)	**(*F* = 3.995E3)
Month	4	**(*F* = 349.581)	**(*F* = 82.360)	**(*F* = 98.175)	**(*F* = 210.688)
Sowing treatment×Month	4	**(*F* = 6.025)	*(*F* = 4.011)	**(*F* = 29.037)	**(*F* = 209.446)

*: *P* < 0.05

**: *P* < 0.001.

**Fig 2:**
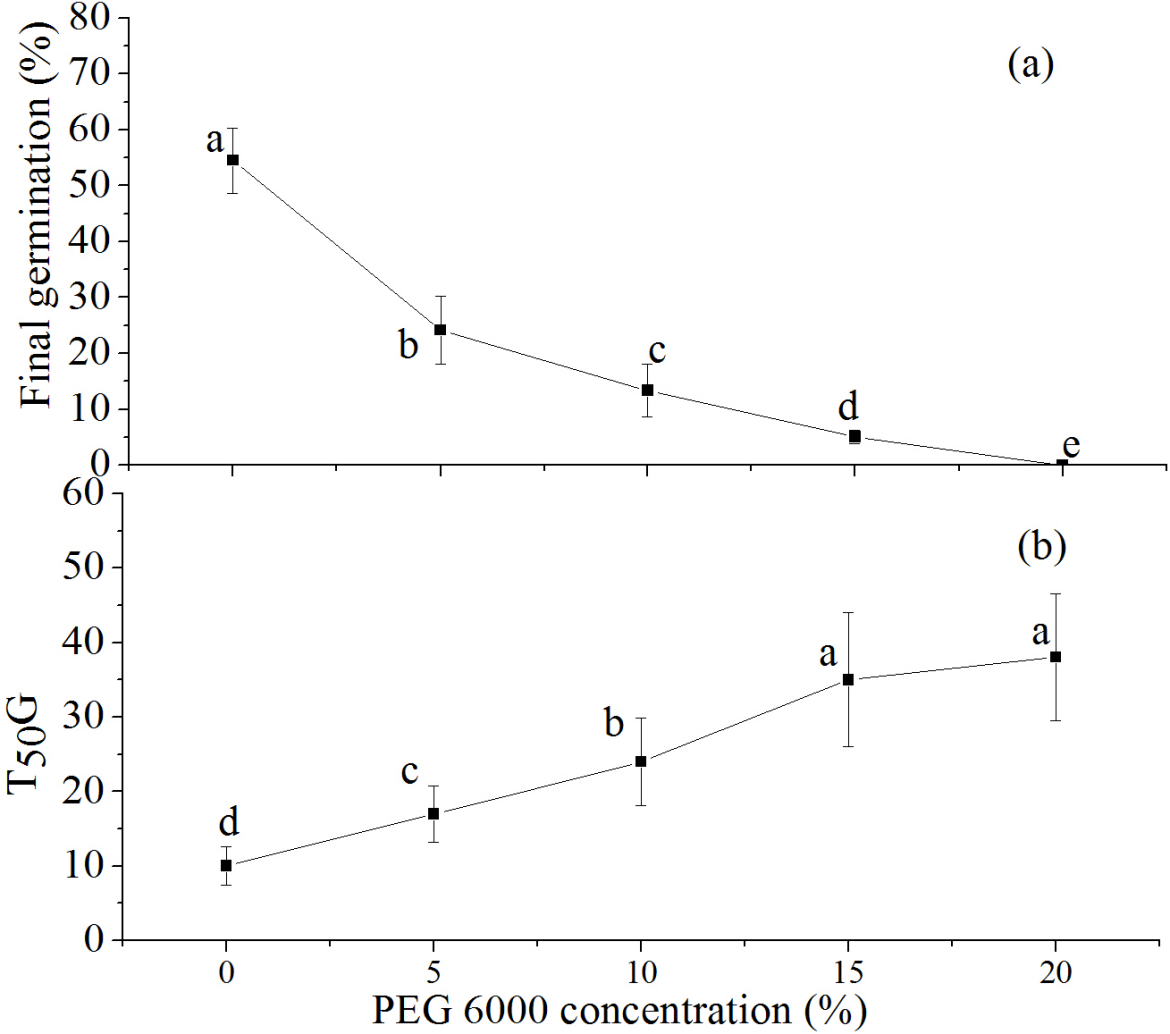
Effects of simulated drought stress on final seed germination rate (a) and time to 50% germination (b) of *Nyssa yunnanensis* seeds.

The SDS experiment on *N*. *yunnanensis* seed germination indicated that the final germination rate declined significantly with the increase in PEG 6000 concentration. It decreased from 54.5% in distilled water (control) to zero at a PEG 6000 concentration of 20% (-5.34 MPa; Fig 2A, *P* < 0.05). Furthermore, the time to 50% germination increased significantly with the increase in concentration of PEG 6000, from 9.67 days in distilled water to 38.2 days at a PEG 6000 concentration of 20% (-5.34 MPa; Fig 2B, *P* < 0.05). Unfortunately, seeds began to germinate and then all died at a PEG 6000 concentration of 20%.

### Seedling survival

Early seedlings survived for the entire experimental period (40 d) up to a water stress of 15% PEG 6000 (-3.25 MPa). At a PEG 6000 concentration of 20% (-5.34 MPa), seedlings perished. Post-seedlings survived for the entire experimental period (90 d) up to a water stress of 1/4 WHC (-4.46 MPa), but perished under the 1/5 WHC (-5.64 MPa) treatment.

### Seedling growth

Results of the *in situ* field experiment revealed that seedling emergence was related to seed germination and was found to be significantly different between sowing treatments and months (Table 1). Seedling emergence lasted from September to November and the emergence rate was 23.89% in pots and 0.85% in field during the first month (Fig 1B). The highest seedling emergence rate was reached in November, which corresponded to 56.61% in the pots and 23.69% in the field. Seedling emergence stopped from the end of November. As found in the germination experiments, the potted treatment had significantly higher seedling emergence than the field treatment.

Seedling survival in the pots was significantly different to that in the field across the different months (Table 1). The seedlings survived in both the pot and field experiments decreased from November (Fig 1C). In the field treatment, the survival rate was 23.69% in November, dropping to 10.50% in December and reaching 0 in January, indicating that all seedlings perished under natural conditions. However, the seedling survival rates in the pot treatment only decreased slightly, retaining 53.00% survival in January.

The SDS experiment on early seedling growth revealed that all growth indices (root length, shoot length and fresh weight) decreased significantly with an increase in PEG 6000 concentrations (Table 2). Compared to the distilled water control, the growth of seedlings decreased by more than four times and fresh weight decreased by seven times at the PEG 6000 concentration of 10% (-1.66 MPa). No significant differences were observed in any of the parameters under PEG 6000 concentrations of 10% and 15% (*P* > 0.05). However, at a PEG 6000 concentration of 20% (-5.34 MPa), all seedlings perished by the end of the experiment.

**Table 2:** Effects of simulated drought stress on early seedling growth of *Nyssa yunnanensis*.

PEG 6000 concentration (%)	Root length (cm)	Shoot length (cm)	Seedling length (cm)	Seedling fresh weight (g)
0	3.04±0.20a	3.12±0.27a	6.16±0.45a	0.07±0.018a
5	0.97±0.22b	1.45±0.19b	2.41±0.23b	0.03±0.010b
10	0.57±0.13c	0.72±0.08c	1.49±0.14c	0.01±0.006c
15	0.29±0.04d	0.28±0.07d	0.57±0.09d	0.01±0.001c
20	0.26±0.06d	0.27±0.04d	0.53±0.06d	0.01±0.001c

Data are mean±SD (n = 6). Values marked by different letters (a, b, c, d) within a column are significantly different at *P* < 0.05. Same as below.

The water-controlled experiment indicated that relative soil water content significantly influenced the post seedling growth of *N*. *yunnanensis*. All growth indices declined with the decrease in relative soil water content (Table 3).

**Table 3:** Effects of water condition on the growth parameters of *Nyssa yunnanensis* seedlings.

Soil moisture level	Relative growth rate	Total leaf area (cm2)	Number of leaves	Height (cm)	Aboveground biomass (g)	Belowground biomass (g)	Total biomass (g·plant-1)	Root/ Shoot ratio	LAR	SLA
1 WHC	17.79±0.93a	111.66±7.97a	20.60±2.19a	51.22±4.22a	7.61±0.88a	4.05±0.54a	11.66±0.98a	0.53±0.05a	197.21±12.58a	302.27±4.101a
1/2 WHC	13.45±1.10b	50.96±5.04b	16.25±1.98b	30.25±3.35bc	3.16±0.51b	1.48±0.19bc	4.64±0.29b	0.47±0.04a	178.62±16.38a	262.38±17.13a
1/3 WHC	10.05±1.53c	36.81±2.38bc	11.20±1.11c	27.75±2.92c	2.07±0.21bc	0.84±0.07c	2.91±0.26b	0.41±0.04a	165.45±10.86a	199.18±12.36b
1/4 WHC	9.19±0.82c	23.15±3.17c	6.75±0.87d	23.66±2.20c	2.04±0.07c	0.76±0.11c	2.80±0.22b	0.37±0.05a	101.81±8.61b	206.30±18.05b
1/5 WHC	8.59±0.49c	21.75±1.96c	5.45±0.59d	20.66±1.85c	1.84±0.24c	0.53±0.07c	2.41±026b	0.32±0.03a	98.44±10.87b	195.76±16.87b

## Discussion

Seed germination and seedling establishment depend primarily on moisture availability [40], soil salinity [17] and phytotoxic molecules [41]. Poor water availability is considered to be one of the principal causes of unsuccessful seedling establishment [42]. Soil moisture is especially critical for seedlings, which are prone to die-off in the dry season [43]. In our *in situ* field experiment, seed germination and seedling establishment were significantly lower under field conditions with naturally available moisture than under potted conditions with a sufficient moisture supply, across all three sites. Furthermore, seed germination, seedling emergence and seedling survival continued to increase until November, corresponding to a soil water potential of -4.30 MPa. Thereafter, seed germination and seedling emergence terminated physiologically in both the potted and field experiments. Seedlings began to perish in Dec. (-5.40 MPa), and had all died -off by Jan. at a soil water potential of -5.60 MPa in the field treatment. This implied that a soil water potential of -5.40 MPa severely limits *N*. *yunnanensis* seedlings. These results support our hypothesis that drought stress hinders seed germination and seedling survival. However, the exact mechanism by which drought stress suppresses *N*. *yunnanensis* regeneration and threatens its survival remains elusive.

Numerous studies have demonstrated that drought stress inhibits seed germination and seedling growth [44–49]. For many plants, the stages of seed germination and early seedling growth are highly sensitive to environmental stresses [50]. The strongest decline in germination has been observed at a high PEG concentration in several studies [51–53]. Similarly, seed germination in *N*. *yunnanensis* was progressively inhibited with increasing PEG 6000 concentrations (-0.01 –-5.34 MPa) in our simulated drought stress (SDS) experiment. Our results indicated that the soil water potentials experienced between Sep. and Jan. (-1.10 –-5.60 MPa) in the field result in water stress, suppressing seed germination.

The early seedling growth parameters were also significantly affected by simulated drought stress. Root length, shoot length and fresh weight decreased significantly with an increase in PEG 6000 concentration (-0.01 –-5.34 MPa), and all the seedlings had perished at a PEG 6000 concentration of 20% (-5.34 MPa). Our results support the idea that seed germination and early seedling growth are impacted by simulated drought stress [54–56] Early seedlings were significantly inhibited when soil water potential was at a PEG 6000 concentration of 18% (-4.29 MPa), corresponding to that observed in the field in Nov. (-4.30 MPa). Results of the water-controlled experiment confirmed these observations and indicated poor drought resistance in *N*. *yunnanensis*. Ten growth indicators of *N*. *yunnanensis* seedlings were significantly inhibited by drought stress (-0.01 –-4.46 MPa) and seedlings perished at 1/5 WHC (-5.64 MPa). The fact that root/ shoot ratio declined with the decrease in relative soil water content is not in accordance with the normal response of plants, which is to increase belowground organ biomass to encourage nutrient and water update under drought conditions. This suggests that *N*. *yunnanensis* seedlings are not able to dynamically adapt to water deficit via responses in morphology. Hence, the implication is that seedlings in field had also been suppressed by drought stress, and a range of soil water potentials from -5.34 MPa to -5.64 MPa are fatal for *N*. *yunnanensis* seedlings. These results also indicated that seed germination and seedling establishment in the field had been suppressed during the seedling establishment period from Sep. to Feb. In accordance with the findings of other plant studies [57–59], low water availability significantly inhibited the growth of *N*. *yunnanensis* seedlings. Conversely, higher soil moisture content culminated in an increase in leaves and larger leaf areas of seedlings, which resulted in a greater photosynthetic rate as well as a higher biomass. When experiencing drought stress, most plants will reduce their aboveground biomass and allocate more resources to their belowground biomass, so as to improve the likelihood of survival by increasing access to water and nutrients [2,14,60–62]. Interestingly, the root/shoot ratio of *N*. *yunnanensis* seedlings declined with the decrease of soil moisture. Therefore, susceptibility to drought stress along with low phenotypic plasticity in a changing environment might be a major factor in the regeneration failure of *N*. *yunnanensis*.

Climate change-induced drought is the most frequent cause of forest regeneration failure [19,42,50]. Global climate change is projected to produce more frequent droughts in many regions of the world [6,12], and can trigger regeneration failure in natural forests [19]. According to the meteorological records of Puwen Forest Farm over the past 55 years, the climate in the natural distribution area of *N*. *yunnanensis* has changed dramatically. There has been a clear decreasing trend in annual mean rainfall and relative humidity, declining by 21.7% and 6.3% respectively from 1960 to 2014, while annual mean temperature has increased by 6.0% (S1 Fig). In the context of decreased precipitation, higher temperatures are associated with an increased occurrence of drought due to higher rates of evapotranspiration. Importantly, decreasing precipitation and increasing temperature from September to February (S2 Fig) intensifies the soil moisture deficit during the key growth phrases of seedling establishment of *N*. *yunnanensis*. It appears that water stress as a result of decreasing rainfall severely influences the integrity of *N*. *yunnanensis* seedlings, especially when autotoxicity is concerned. According to Zhang et al. [30], the autotoxic effects of *N*. *yunnanensis* might be concentrated under lower water potential conditions, thereby further hindering seed germination and seedling establishment. Thus, climatic change-induced drought may be primarily responsible for the regeneration failure of *N*. *yunnanensis*, resulting in a decline in population size. In addition to the changes in global climate trends, the microclimate changes in natural habitat of *N*. *yunnanensis* are influenced by local production activities. For instance, many local natural forests have been replaced by rubber, coffee, tea and other plantations [30]. Therefore, the conservation strategies for *N*. *yunnanensis* should encompass physiological or adaptive obstacles in seedling establishment, preventing habitat loss as a result of human disturbances, as well as the maintenance of microclimate stability.

## Conclusion

Results from our field and laboratory experiments revealed that climate change-induced drought stress impacts the natural forest habitat of *N*. *yunnanensis*, suppressing seed germination and seedling growth in the process of natural regeneration, threatening the survival of the species. It was discovered that soil water potentials ranging from -5.34 MPa to -5.64 MPa are fatal for *N*. *yunnanensis* seedlings, and can probably be attributed to the simultaneous impacts of water deficit and aggravated autotoxicity. Effective conservation measures, such as habitat restoration and microclimate improvement, should be considered in the conservation strategy.

## Supporting information

S1 Fig Trend in annual mean rainfall (a), temperature (b) and relative humidity (c) over the past 55 years at Puwen Experimental Forest Farm.
(TIF)

S2 Fig Monthly mean temperature and precipitation in a year at the study site. (TIF)
