# Supplementary figures and images for: Climate change-induced water stress suppresses the regeneration of the critically endangered forest tree Nyssa yunnanensis
Source: PLoS One. 2017 Aug 1;12(8):e0182012. doi: 10.1371/journal.pone.0182012 (PMC5538672; doi:10.1371/journal.pone.0182012)

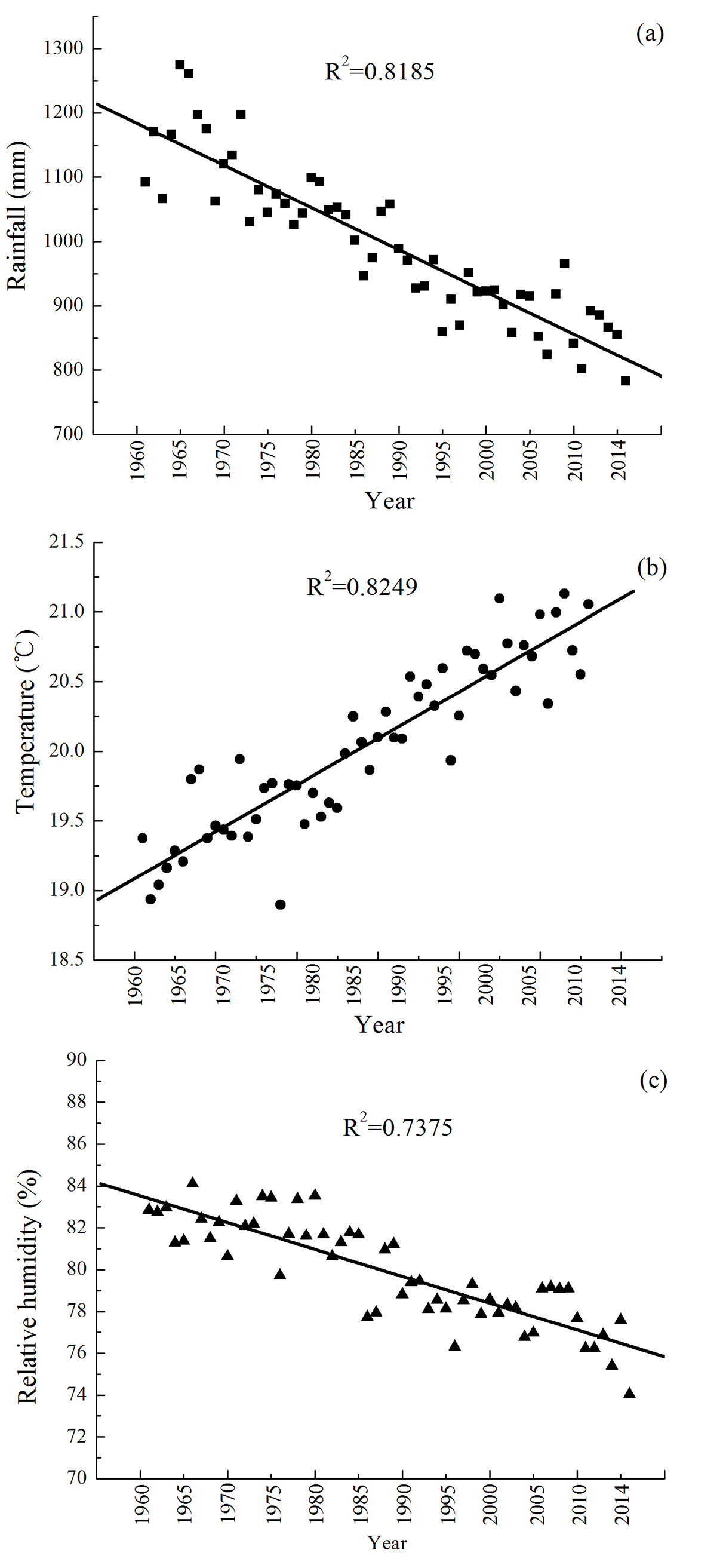

Supplement: S1 Fig — Trend in annual mean rainfall (a), temperature (b) and relative humidity (c) over the past 55 years at Puwen Experimental Forest Farm. (TIF) [file pone.0182012.s001.tif]

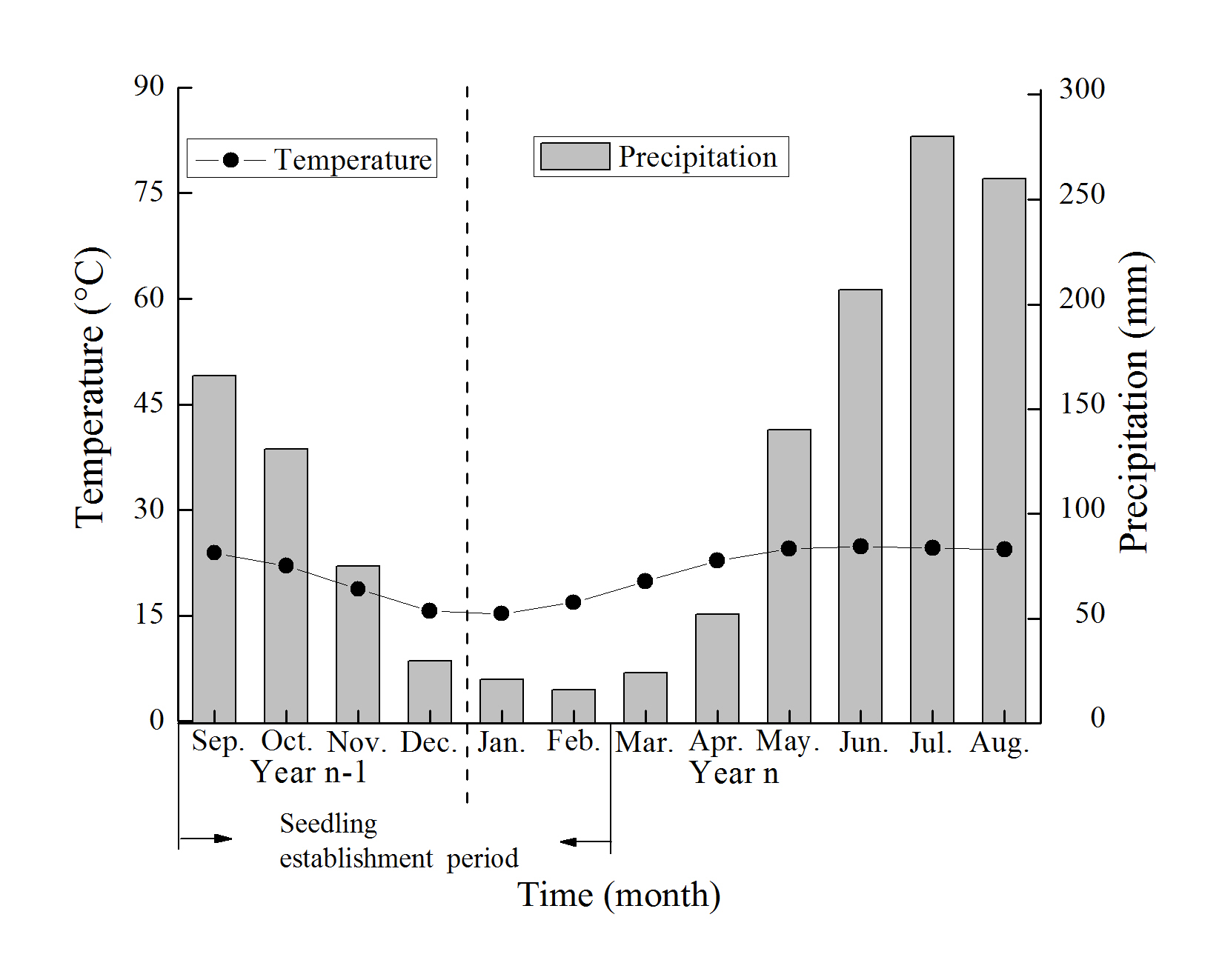

Supplement: S2 Fig — (TIF) [file pone.0182012.s002.tif]
